# Does a suggested diagnosis in a general practitioners’ referral question impact diagnostic reasoning: an experimental study

**DOI:** 10.1186/s12909-022-03325-7

**Published:** 2022-04-08

**Authors:** J. Staal, M. Speelman, R. Brand, J. Alsma, L. Zwaan

**Affiliations:** 1 Erasmus University Medical Center Rotterdam, Institute of Medical Education Research, Rotterdam, the Netherlands; 2 Faculty of Medical Sciences, Erasmus University Medical Center Rotterdam, Rotterdam, the Netherlands; 3 Intensive Care Unit, Haaglanden Medical Center Den Haag, The Hague, the Netherlands; 4 Department of Internal Medicine, Erasmus University Medical Center Rotterdam, Rotterdam, the Netherlands

**Keywords:** Diagnostic error, Clinical reasoning, Cognitive bias, Patient safety

## Abstract

**Background:**

Diagnostic errors are a major cause of preventable patient harm. Studies suggest that presenting inaccurate diagnostic suggestions can cause errors in physicians’ diagnostic reasoning processes. It is common practice for general practitioners (GPs) to suggest a diagnosis when referring a patient to secondary care. However, it remains unclear via which underlying processes this practice can impact diagnostic performance. This study therefore examined the effect of a diagnostic suggestion in a GP’s referral letter to the emergency department on the diagnostic performance of medical interns.

**Methods:**

Medical interns diagnosed six clinical cases formatted as GP referral letters in a randomized within-subjects experiment. They diagnosed two referral letters stating a main complaint without a diagnostic suggestion (control), two stating a correct suggestion, and two stating an incorrect suggestion. The referral question and case order were randomized. We analysed the effect of the referral question on interns’ diagnostic accuracy, number of differential diagnoses, confidence, and time taken to diagnose.

**Results:**

Forty-four medical interns participated. Interns considered more diagnoses in their differential without a suggested diagnosis (M = 1.85, SD = 1.09) than with a suggested diagnosis, independent of whether this suggestion was correct (M = 1.52, SD = 0.96, d = 0.32) or incorrect ((M = 1.42, SD = 0.97, d = 0.41), χ2(2) =7.6, *p* = 0.022). The diagnostic suggestion did not influence diagnostic accuracy (χ2(2) = 1.446, *p* = 0.486), confidence, (χ2(2) = 0.058, *p* = 0.971) or time to diagnose (χ2(2) = 3.128, *p* = 0.209).

**Conclusions:**

A diagnostic suggestion in a GPs referral letter did not influence subsequent diagnostic accuracy, confidence, or time to diagnose for medical interns. However, a correct or incorrect suggestion reduced the number of diagnoses considered. It is important for healthcare providers and teachers to be aware of this phenomenon, as fostering a broad differential could support learning. Future research is necessary to examine whether these findings generalize to other healthcare workers, such as more experienced specialists or triage nurses, whose decisions might affect the diagnostic process later on.

**Trial registration:**

The study protocol was preregistered and is available online at Open Science Framework (https://osf.io/7de5g).

**Supplementary Information:**

The online version contains supplementary material available at 10.1186/s12909-022-03325-7.

## Introduction

Diagnostic errors are a large burden on patient safety. It is estimated that a majority of patients will suffer at least one diagnostic error during their lifetime, sometimes with devastating consequences [1–3]. Diagnostic errors are defined as “the failure to establish and/or communicate an accurate and timely explanation of the patient’s health problem(s)” [1]. Most of these errors are thought to be preventable [1, 4]. In order to develop successful interventions, it is crucial to understand the underlying causes of diagnostic errors.

Physicians working in the ED often use clinical information (e.g., symptoms, examination findings, or test results) from patient referral letters in diagnostic decisions. The referral process is vulnerable to breakdowns in the process itself [5–7] and can also be influenced by flaws in the cognitive processes of the involved physicians. Flawed cognitive processes are seen as an important cause of diagnostic errors. These cognitive errors are often explained using dual process theory (DPT), which hypothesizes that reasoning consists of a non-analytical and fast System 1, and an analytical and more deliberate System 2 [8, 9]. Errors in System 1 are often ascribed to cognitive biases [10], which are introduced into the reasoning process because of incorrect assumptions or missed information. Errors in System 2, on the other hand, are often ascribed to knowledge deficits [11, 12]. In a clinical context, cognitive errors could cause physicians to be influenced by incorrect information from another physician or to incorrectly interpret clinical information, which could ultimately result in diagnostic errors. Especially emergency medicine physicians are prone to such errors, due to domain specific factors such as complex decision making under time pressure and high uncertainty [13, 14].

Previous studies show that clinical information can indeed influence diagnostic accuracy. For example, accurate clinical information improved physicians’ true positive rates in radiology and test reading [15–17], whereas inaccurate clinical information reduced diagnostic accuracy [18] and even biased physicians’ diagnostic reasoning towards incorrect working diagnoses suggested by the clinical information [19]. This effect was found for medical students as well as for experienced physicians [20]. However, it remains unclear via which underlying processes clinical information can impact diagnostic accuracy. For example, accuracy could decrease due to overconfidence, a limited differential diagnosis, or because physicians do not spend enough time on a case.

In this experimental study, we examined the effect of a general practitioner’s (GPs) suggested diagnosis when they refer a patient from primary care (i.e., general practice) to secondary care (i.e., the ED) on the diagnostic performance of medical interns. The suggested diagnosis in a GPs referral question could be correct or incorrect, or did not contain a diagnostic suggestion at all (control group). We studied diagnostic performance in terms of diagnostic accuracy, and expanded on previous research by adding measures of differential diagnosis, confidence, and time spent on a case.

We expected that the suggested diagnosis in a GPs referral letter would cause interns to more often follow the suggested diagnosis than when no suggested diagnosis was provided (control condition). We hypothesized that this would also be true if the suggestion was incorrect. Furthermore, we hypothesized that both a correctly and an incorrectly suggested diagnosis would reduce the number of differential diagnoses considered and decrease the time spent to diagnose compared to the control group. Lastly, we expected that confidence in the most likely diagnosis would increase relative to the control group.

## Methods

### Participants

Medical interns associated with the Erasmus University Rotterdam (EUR) and the Erasmus University Medical Center (Erasmus MC) were invited to participate. Participants were eligible if they had completed their clinical rotation in internal medicine. Using G-power 3.1.9.7 [21] a sample size of 36 participants was estimated for a repeated measures analysis of variance (ANOVA) with a medium effect size based on Meyer et al. [20], a power of 0.95, and an alpha level of 0.05.

### Design

A randomized within-subjects experiment was conducted in which each participant diagnosed six cases in three conditions. Participants were presented with two cases stating the patient’s main complaint without a diagnostic suggestion, two cases with a correct diagnostic suggestion, and two cases with an incorrect diagnostic suggestion. Case order and condition were randomised through partial counterbalancing using a Latin square (Additional file 1).

### Materials

Six fictional cases were developed by an expert internist (JA), a medical doctor (RB) and a medical student (MS) and were piloted by 6 medical doctors specialized in primary care or internal medicine. Each case had one correct diagnosis and one plausible (but incorrect) alternative diagnosis (Table 1). All cases were formatted to look like genuine referral letters from a primary care physician (Additional file 2) and were presented in Dutch. Participants used their own device (laptop or mobile phone) to access the survey in which the cases were presented (Additional file 3).

**Table 1:** Overview of the primary complaint, the alternative (incorrect) diagnostic suggestion and the correct diagnostic suggestion

	No suggestion	Correct suggestion	Incorrect suggestion
Case 1	Abdominal pain	Ovarian torsion	Appendicitis
Case 2	Painful leg	Erysipelas	Deep vein thrombosis
Case 3	Dyspnea	Heart failure	Pneumonia
Case 4	Epigastric pain	Pancreatitis	Cholecystitis
Case 5	Retrosternal pain	Peptic ulcer	Myocardial infarction
Case 6	Colic	Gallstone	Kidney stone

### Procedure

Participants read an information letter and signed informed consent before participation. In order to study the effect of the manipulated referral question, the study’s purpose was not fully disclosed to participants in advance. Instead, participants were told that we wanted to pilot the difficulty level of several clinical cases that were to be used for education. Participants diagnosed the six cases and after every case, they were asked to provide their most likely diagnosis (free text) and to rate their confidence in this diagnosis (0 = no confidence, 10 = very confident). The time that participants took to complete each case was registered upon submitting the diagnosis. After diagnosing all cases, participants were shown the case again and were asked to provide differential diagnoses for each of the cases. The differential diagnosis was elicited after all six cases were diagnosed to prevent the possible induction of reflective reasoning, which could reduce the effect of our manipulation [22]. Finally, they were asked to provide their demographic information and what they thought the real goal of the study was.

### Outcome measures

Diagnostic accuracy was quantified by scoring the most likely diagnosis as either correct (1 point), partially correct (0.5 points) or incorrect (0 points). A diagnosis was scored as correct if participants mentioned the correct diagnosis or a different term for the same diagnosis. Closely adjacent diagnoses were also given full points (i.e., the correct diagnosis was pancreatitis and the participant mentioned acute pancreatitis). A diagnosis was scored as partially accurate if the participant captured an element of the diagnosis, but left out another core element (i.e., the correct diagnosis was peptic ulcer and the participant mentioned only ulcer). Any other diagnoses were scored as incorrect and did not receive any points. Scoring was performed independently by a medical doctor (RB) and a medical student (MS). If there was a discrepancy, this was solved via discussion with an expert internist (JA) as the third rater. Confidence in the most likely diagnosis was measured on a scale from 0 to 10 as self-reported by the participant. Time spent to diagnose was measured in seconds and automatically recorded by the survey software (Qualtrics). Based on the time taken to diagnose in the pilot, any entrees that took less than 25 s were considered not realistic and therefore excluded. Lastly, differential diagnosis was measured as the number (count) of alternative diagnoses given in a free text box.

#### Demographics

We measured the following demographic information: age, sex, months spent in the clinical phase, current internship, and specialism of interest. Additionally, we performed a manipulation check by asking participants to guess the study’s goal.

### Statistical analysis

According to the Kolmogorov-Smirnov test, the data were not normally distributed. Therefore, a within-subjects Friedman’s ANOVA was performed to test if the referral question (within-subjects factor) impacted students’ diagnostic performance. Separate Friedman’s ANOVAs were performed for mean diagnostic accuracy, differential diagnosis, confidence, and time to diagnose a case, which were averaged per participant per condition. Additionally, differential diagnosis, confidence, and time to diagnose for correct and incorrect most likely diagnoses were compared using the Wilcoxon signed rank test. If a Friedman’s ANOVA was significant, post-hoc tests were performed using individual Wilcoxon signed rank tests. A *p*-value of < 0.05 was considered statistically significant. Statistical analyses were performed using SPSS statistical software, version 25 for Windows (IBM Corp., Armonk, New York).

## Results

Forty-four out of the total ninety-seven participants (45%) completed the experiment, 5 (5%) quit halfway through the study and 48 (50%) did not get past the initial instructions. Of the 44 students who completed the study, five participants were excluded based on the cut-off value for time to diagnose (< 25 s), leaving 39 participants in the main analysis. For the analysis of the differential diagnosis, an additional five students were excluded because they did not provide a differential diagnosis for any of the cases. Demographics were available for 38 participants. Thirty-one participants (82%) were female. On average, participants were 24 years (SD = 1) old and had spent 21 months (SD = 8) in the clinical phase. Age, sex, and months in the clinical phase did not moderate accuracy, number of differential diagnoses, confidence or time to diagnose (all *p* > 0.05) and thus, did not need to be corrected for.

### Manipulation check

Seven out of the 39 participants (17.94%) correctly identified the study’s goal. Despite this, their performance (diagnostic accuracy: M = 0.50, SD = 0.32) was similar to participants who did not identify the study’s goal (diagnostic accuracy: M = 0.51, SD = 0.32). Therefore, all participants were analysed as one group.

### Main analysis

The diagnostic suggestion did not influence diagnostic accuracy, χ^2^(2) = 1.45, *p* = 0.486, but did impact the number of differential diagnoses generated, χ^2^(2) = 7.60, *p* = 0.022. Interns considered significantly more diagnoses when they did not receive a diagnostic suggestion compared to when they did, which resulted in a small effect size compared to both correct suggestions (d = 0.32) and incorrect suggestions (d = 0.41). Confidence, χ^2^(2) = 0.06, *p* = 0.971 and time to diagnose, χ^2^(2) = 3.13, *p* = 0.209, did not differ significantly depending on the referral question. Descriptive data are reported in Table 2.

**Table 2:** Mean (M) and standard deviation (SD) for accuracy, differential diagnosis, confidence and time to diagnose

	N	No suggestion	Correct suggestion	Incorrect suggestion
Accuracy, M (SD)	39	0.46 (0.34)	0.57 (0.27)	0.51 (0.35)
Differential diagnosis, M (SD)	33	1.85 (1.09)	1.52 (0.96)	1.42 (0.97)
Confidence, M (SD)	39	6.23 (1.23)	6.21 (1.00)	6.36 (1.13)
Time to diagnose, M (SD)	39	120.21 (55.87)	133.79 (81.87)	140. 40 (74.78)

### Exploratory analyses

#### Accuracy per case

The effect of diagnostic suggestion on diagnostic accuracy was not significant overall, but there was substantial variation between the cases used (Table 3). Notably, accuracy was descriptively higher for a correct diagnostic suggestion in case 1 (50%) and case 5 (63.64%) compared to an incorrect diagnostic suggestion (case 1: 13.33%; case 5: 53.85%) or no diagnostic suggestion (case 1: 16.67%; case 5: 26.67%). Conversely, accuracy was descriptively lower for the correct diagnostic suggestion for case 3 (68.76%) and case 6 (43.75%) compared to an incorrect diagnostic suggestion (case 3: 90.91%; case 6: 75%) or no diagnostic suggestion (case 3: 100%; case 6: 72.72%).

**Table 3:** The number of responses and percentage (%) of correct responses per case

Condition	Case 1	Case 2	Case 3	Case 4	Case 5	Case 6	Total
No suggestion	2/12 (16.67%)	4 /13 (30.77%)	12/12 (100%)	7/15 (46.67%)	4/15 (26.67%)	8/11 (72.73%)	37/78 (47.43%)
Correct suggestion	6/12 (50%)	6/11 (54.55%)	11/16 (68.76%)	9/12 (75%)	7/11 (63.64%)	7/16 (43.75%)	46/78 (58.97%)
Incorrect suggestion	2/15 (13.33%)	11/15 (73.33%)	10/11 (90.91%)	5/12 (41.67%)	7/13 (53.85%)	9/12 (75%)	44/78 (56.41%)
Total correct per case	10/39 (25.64%)	21/39 (53.85%)	33/39 (84.62%)	21/39 (53.85%)	18/39 (46.15%)	24/39 (61.54%)	127/234 (54.27%)

#### Correct and incorrect diagnosis

The number of diagnoses considered in the differential diagnosis did not differ between participants who gave a correct diagnosis (M = 1.54, SD = 1.22) and participants who gave an incorrect diagnosis (M = 1.59, SD = 1.22), T = 1407.00, *p* = 0.767. The time that participants spent to diagnose cases also did not differ between correct and incorrect diagnoses (correct: M = 129.29, SD = 104.07; incorrect: M = 136.57, SD = 88.27, T = 2620.00 *p* = 0.322).

#### Confidence

Participants were more confident when their most likely diagnosis was correct (M = 6.51, SD = 0.97), compared to when it was incorrect (M = 6.00, SD = 1.03), T = 1592.00, *p* = 0.006, d = 0.51. This did not differ based on the diagnostic suggestion, χ2 (3) = 4.29, *p* = 0.232 (Table 2).

## Discussion

This study examined the effect of clinical information in the form of a diagnostic suggestion in a GPs referral letter on the diagnostic performance of medical interns. Contrary to our hypotheses, we found no effect of diagnostic suggestion on accuracy, confidence, or time taken to diagnose. Diagnostic suggestions did, however, affect the number of diagnoses participants considered in their differential diagnosis. They considered more diagnoses when the referral letter did not contain any suggestion compared to when either a correct or incorrect suggestion was presented. Exploratory analyses further suggested a positive correlation between accuracy and confidence.

Research on the effect of clinical information on test reading has shown that diagnostic suggestions can bias physicians towards the suggested diagnosis, decreasing diagnostic accuracy if the suggestion was incorrect [18, 19]. The interns in the current study, however, were able to overcome the potential bias of an incorrect suggestion, as their accuracy did not decrease. This contrast to previous studies might be explained by the relative inexperience of our participants. It is suggested that inexperienced physicians rely more on analytical reasoning than on non-analytical reasoning, as they have not accumulated enough previous experiences to rely on pattern recognition [23]. Reliance on analytical thinking could result in a more conscious approach to diagnosis, possibly making our participants more vigilant for information in the case that conflicted with the suggestion. Such an approach would make participants less likely to be biased by the suggestion as analytical approaches such as deliberate reflection have been shown to reduce diagnostic errors due to biases [22]. This possibility is supported by our finding that confidence was higher when participants were correct: they seemed capable of estimating how valid their diagnoses were, which fits the profile of analytical reasoning.

Although overall diagnostic accuracy was not affected by the type of diagnostic suggestion, exploratory analyses suggested there were differences at case-level. Specifically, our findings indicated that depending on the case, correct diagnostic suggestions could either be beneficial or detrimental to accuracy (Table 3). These differences were descriptive and not statistically significant, but provide considerations for future research. In two cases where less than 50% of participants were correct when receiving no diagnostic suggestion, accuracy improved when they received the correct suggestion. In this scenario, the correct suggestion could possibly compensate for gaps in knowledge by suggesting a diagnosis that the participant otherwise would not have considered [11, 12]. For example, in the first case interns were likely more familiar with appendicitis (the alternative incorrect diagnosis) than with ovarian torsion (the correct diagnosis). The correct suggestion might have prevented them from missing the less prevalent diagnosis and allowed them to suggest the correct diagnosis instead. However, in two other cases accuracy descriptively decreased when a correct diagnostic suggestion was considered. This could indicate that knowledge gaps should be acknowledged before a suggestion can be beneficial. For example, if the incorrect diagnosis seems likely, participants might still choose to reject the correct suggestion. All in all, perhaps the effect of diagnostic suggestions depends on the case diagnosis, participant’s prior knowledge, and their willingness to consider suggestions.

The type of diagnostic suggestion did impact interns’ differential diagnosis: just providing a suggested diagnosis, either correct or incorrect, reduced the number of diagnoses considered. This is consistent with Meyer et al. [20] who showed that an a priori diagnosis, regardless of whether this diagnosis was correct or not, led to fewer questions asked during history taking and a less systematic assessment of differential diagnoses. Failure to consider the correct diagnosis is an important cause of diagnostic error [24]. It is vital that the correct diagnosis is at least considered in the differential diagnosis, even if it is not considered as the most likely diagnosis. The importance of the differential diagnosis is associated with the dynamic nature of diagnostic reasoning. If the course of a disease changes, it will be easier to consider another diagnosis that is already included in the differential diagnosis. But although our diagnostic suggestions did reduce the number of differential diagnoses considered, they did not decrease diagnostic accuracy. Future research should examine whether this reduction in differential diagnoses results in a qualitatively worse differential diagnosis, or conversely if it produces a more specific and efficient differential diagnosis without a reduction in accuracy. Though it is difficult to make practical recommendations based on the current results, we suggest it might be valuable for education to have interns practice diagnosing cases without a diagnostic suggestion as this can allow them to foster a broader differential diagnosis. Additionally, educators could vary between using cases with and without diagnostic suggestions, so that interns can practice with both scenarios and might perhaps learn to overcome possible negative influences or benefit from possible positive influences of suggested diagnoses. For example, interns could be trained using methods such as deliberate reflection, which promote the generation of multiple differential diagnoses and considering information that increases or reduces the likelihood of the differential diagnoses [25]. Such teaching methods could be incorporated into problem-based or case-based learning, where it could let interns practice with creating appropriate differential diagnoses for individual cases.

The current study had several strengths and limitations. Because of the experimental within-subjects design with randomized presentation of the cases and diagnostic suggestions, it was possible to isolate the effect of the diagnostic suggestion. Furthermore, this study had a high power due to the within-subjects design. However, the experimental design also poses a limitation, as we could not replicate the time constraints and high level of uncertainty present in clinical practice. Additionally, the current findings are limited in their generalizability to practice, first because we included relatively inexperienced interns and second because we recruited participants by spreading the survey link online. The latter makes it difficult to calculate a response rate, as we cannot know how many interns saw the link but did not click it. This could limit the external validity of our sample. A related limitation is the high attrition rate (53/97); however, of the interns who started diagnosing cases only 5 failed to complete the experiment. Therefore it seems that participant attrition is not linked to the experiment itself, but rather to motivational factors such as a lack of compensation or personalized recruitment. Future research should replicate the current findings and investigate how diagnostic suggestions affect primary to secondary care referral in clinical practice and in more experienced physicians. Furthermore, this study did not consider the impact of diagnostic suggestions on some steps in the diagnostic process, such as ordering and interpreting investigations, due to practical considerations. Future studies should also consider how diagnostic suggestions impact other steps in the diagnostic process. Finally, the current study only focused on diagnostic suggestions from GPs: future studies could expand to include suggestions from other medical professionals, for example clinicians within the same department as the diagnostician, or from patients themselves, as these suggestions might be given different weights and could differentially affect diagnostic performance.

In conclusion, diagnostic suggestions can reduce the number of diagnoses considered in the differential diagnosis of medical interns. Other aspects of diagnostic performance, namely interns’ diagnostic accuracy, confidence, and time to diagnose, were not affected. Healthcare providers should be aware of this phenomenon in order to limit unwanted effects. When training medical students in clinical reasoning, one could avoid diagnostic suggestions in order to train students in broad differential thinking. Considering the fact that various professionals are involved with the work-up in the ED, future research should repeat the experiment in other groups of professionals, such as medical specialists and triage nurses.

## Supplementary Information

**Additional file 1: Table 1.** Partial randomisation of referral questions and clinical cases using a Latin square.

**Additional file 2.** Example case 1: correct referral question.

**Additional file 3.** Survey questions.

## Data Availability

The study protocol was preregistered and is available online at Open Science Framework (https://osf.io/7de5g).

## Abbreviations

ANOVA: Analysis of variance
DPT: Dual process theory
ED: Emergency department
EUR: Erasmus University Rotterdam
Erasmus MC: Erasmus University Medical Center
GP, GPs: General practitioner, general practitioners
