# Supplementary material for: Does a suggested diagnosis in a general practitioners’ referral question impact diagnostic reasoning: an experimental study
Source: BMC Med Educ. 2022 Apr 8;22:256. doi: 10.1186/s12909-022-03325-7 (PMC8991944; doi:10.1186/s12909-022-03325-7)
Supplement: Supplementary file 1 — Additional file 1: Table 1. Partial randomisation of referral questions and clinical cases using a Latin square. [file 12909_2022_3325_MOESM1_ESM.docx]

**Additional file 1**

*Table 1.* Partial randomisation of referral questions and clinical cases using a Latin square.

| Order | Broad | Broad | Specific congruent | Specific congruent | Specific incongruent | Specific incongruent |
| --- | --- | --- | --- | --- | --- | --- |
| A | Case 1 | Case 2 | Case 6 | Case 3 | Case 5 | Case 4 |
| B | Case 2 | Case 3 | Case 1 | Case 4 | Case 6 | Case 5 |
| C | Case 3 | Case 4 | Case 2 | Case 5 | Case 1 | Case 6 |
| D | Case 4 | Case 5 | Case 3 | Case 6 | Case 2 | Case 1 |
| E | Case 5 | Case 6 | Case 4 | Case 1 | Case 3 | Case 2 |
| F | Case 6 | Case 1 | Case 5 | Case 2 | Case 4 | Case 3 |
