# Supplementary material for: Does a suggested diagnosis in a general practitioners’ referral question impact diagnostic reasoning: an experimental study
Source: BMC Med Educ. 2022 Apr 8;22:256. doi: 10.1186/s12909-022-03325-7 (PMC8991944; doi:10.1186/s12909-022-03325-7)
Supplement: Supplementary file 2 — Additional file 2. Example case 1: correct referral question. [file 12909_2022_3325_MOESM2_ESM.docx]

**Additional file 2 – Example case 1: correct referral question.**

**Referral letter for ED**

Sender Patient

Name: ______________________ Name: ______________

AGB-code: ______________________ Date of birth: ______________

Organisation: ______________________ Citizen service nr.: ______________

______________________ Address: ______________

City: ______________

Org. AGB-code: ______________________ Phone number: ______________

Address: ______________________ Health insurer: ______________

City: ______________________

Phone: ______________________ Patient ID: ______________

Peer consultation: ______________________ Healthcare institution:______________

Referral

Date: ______________________ Name of product: ______________

ZD-number: ______________________ Waiting time: ______________

Organisation: ______________________ Care question: ______________

Address: ______________

Residence: ______________

**Core part** 25-09-2020 14:32

Dear colleague,

*Reason for referral* I hereby refer the following patient (details below) with complaints of pain in the abdomen, ovarian torsion?

*Journal* **Patient contact 23-05-2019**

Patient has pain in the abdomen since yesterday evening, mostly pain in the lower abdomen. Also a bit nauseous. She didn’t vomit until now. Last stool was yesterday afternoon. No pain during movement.

Last menstruation was two weeks ago. No trauma prior to this pain.

PE: very uncomfortable and painful patient, temp. 37.2(ear), heartrate 103.

Calm peristalsis, normal tympany, active defense, possible to palpate the abdomen with deep breathing, tenderness in particular in the right lower quadrant of the abdomen.

Further investigation:

HCG negative

CRP 35

*Medical history* **Medical history**

11-02-19 Insertion of IUD – ultrasound: good position, benign adnexal enlargement 5 cm

15-10-18 Allergic reaction/allergy - dust mite, cat, dog

12-03-15 Mononucleosis infectiosa

22-09-13 Eczema – Dermovate lotion

*Actual medication* Dermovate lotion

Kind regards,

______________
