# Supplementary material for: Does a suggested diagnosis in a general practitioners’ referral question impact diagnostic reasoning: an experimental study
Source: BMC Med Educ. 2022 Apr 8;22:256. doi: 10.1186/s12909-022-03325-7 (PMC8991944; doi:10.1186/s12909-022-03325-7)
Supplement: Supplementary file 3 — Additional file 3. Survey questions. [file 12909_2022_3325_MOESM3_ESM.docx]

**Additional file 3 – Survey questions**

First, participants are asked to evaluate 6 clinical cases. For two cases, no diagnostic suggestion is provided (but only the patients’ main complaint); for two cases, a correct diagnostic suggestion is provided; and for two cases, an incorrect diagnostic suggestion is provided. For each case participants are asked to answer the following questions:

1. What is the most likely diagnosis?
2. How confident are you in your diagnosis? (on a scale form 0-10, no confidence to very confident)

After completing all cases, participants are again shown the case and are asked:

1. Have you considered any other diagnosis? If yes, please list these diagnoses.

Then, participants are asked for relevant demographic information:

1. How old are you?
2. What is your sex?
3. How many months have you spent in your clinical phase?
4. What is the department of your current internship? In case you are currently not following an internship, please name your last completed internship.
5. Which specialism do you want to, or will you, practice after residency?

And we asked participants to guess the goal of the study to check our manipulation:

1. At the start of this study, we informed you that we aimed to evaluate the included cases as exam materials. In addition to that, we also have a secondary goal. Do you have any idea what this goal could be?

Finally, participants are asked to leave their e-mail address so that they can receive information on the study’s outcomes after the study has been completed.

1. If you would like to receive information about the study and its outcomes (and your own performance) when the study has concluded, please leave your email address.
